# Individual and Environmental Predictors of Age of First Intercourse and Number of Children by Age 27

**DOI:** 10.3389/fpsyg.2020.01639

**Published:** 2020-07-08

**Authors:** Wojciech Ł. Dragan, John E. Bates, Jennifer E. Lansford, Kenneth A. Dodge, Gregory S. Pettit

**Affiliations:** ^1^Faculty of Psychology, University of Warsaw, Warsaw, Poland; ^2^Department of Psychological and Brain Sciences, Indiana University Bloomington, Bloomington, IN, United States; ^3^Center for Child and Family Policy, Sanford School of Public Policy, Duke University, Durham, NC, United States; ^4^Department of Human Development and Family Studies, College of Human Sciences, Auburn University, Auburn, AL, United States

**Keywords:** personality, number of children, reproductive behavior, IQ, SES

## Abstract

Reproductive behavior characteristics may be influenced by both social and individual factors. Recent studies have revealed that personality traits might be related to reproductive characteristics in adulthood. Little is known about potential mediators or moderators of relations between personality and reproductive behavior. The present study examines the relation between personality traits measured in early adolescence and the number of children people have by age 27, with an attempt to identify moderation and mediation effects. We used data from the longitudinal cohort (*N* = 585) collected as a part of the Child Development Project. Personality was measured with the use of Lanthier’s Big Five Personality Questionnaire. Results from regression analyses and structural equation models showed that four of the five personality traits (except extraversion) were related to the number of children individuals had by age 27, and these associations were mediated by the age of first intercourse and participants’ familial and educational plans. We also identified moderation effects of IQ and SES both on the associations of personality traits with mediators and the number of children by age 27.

## Introduction

One of the consequences of modernization processes taking place in Western societies is a shift in the importance of motives and factors influencing the decision to have a child—those based on social pressure or biology are becoming less important than ones based on the preferences of an individual (van de Kaa, 2001). The latter may include preferences for different forms of contraceptives, but also more dispositional features like personality traits. Emerging evidence indicates the role of personality in shaping reproductive behavior characteristics. In a series of studies (Jokela et al., 2009, 2011) revealed that traits related to emotion sensitivity and reactivity such as neuroticism and harm avoidance are negatively associated with the number of children adults have. In contrast, extraversion and other related traits (e.g., sociability) seem to be positively related to the number of children people have (Dijkstra and Barelds, 2009; Jokela et al., 2009). Moreover, Jokela et al. (2011) found that openness was a negative predictor of the number of children. They also found that high agreeableness and low conscientiousness predicted having a larger number of children. However, some studies have reported no association of personality variables, including neuroticism (Dijkstra and Barelds, 2009) and extraversion (Nettle, 2005) with the number of children. Although the literature so far does suggest that personality may be associated with reproduction indicators, the relation between personality and fertility patterns is still understudied.

Miller (1994) proposed a model describing how dispositional traits might be translated into specific intentions related to procreation. The first two stages of Miller’s model—the formation of traits and the translation of traits into desires—are of particular importance for the current study. Some studies (Miller, 1992) have shown that indeed personality differences in adulthood are related to reproductive desires and intentions. However, it is unclear how this relation is established during childhood and adolescence. In previous longitudinal studies, personality was measured in late adolescence, at the earliest (Jokela et al., 2009). According to Miller (1994) the formation of traits related to procreation involves the interaction of biologically-based characteristics of an individual with life experiences. Much research has shown that ethnicity and socioeconomic status might impact fertility rates (Sweeney and Raley, 2014). Another possible factor influencing reproductive patterns is the experience of stressful life events (SLE). Belsky et al. (1991) hypothesized that psychosocial stress in the childhood family environment may accelerate sexual activity. This, in turn, may lead to teenage pregnancy and parenthood, and subsequently, a higher number of children. Interestingly, early sexual activity has been reported to be associated with extraversion (Martin et al., 1977). Moreover, sexual behavior in adolescence is related to family and educational plans (Pearson et al., 2012) which themselves may be affected by personality characteristics. Elder and MacInnis (1983) showed that girls who described themselves as “unhappy and lacking friends” were less family oriented and more career oriented. Those with conscientiousness-related traits were also more interested in career achievements. The interaction of family setting and child’s personality in reproductive patterns seems to be an exemplification of Miller (1994) translation of traits into specific strategies and desires.

There is a considerable amount of data showing the impact of IQ on reproduction patterns (Retherford and Sewell, 1989). High intelligence might serve as a protective factor in sexual timing. Halpern et al. (2000) discovered that adolescents who scored high on the Peabody Picture Vocabulary Test were less likely to engage in sexual activity than their average peers. This relation is usually interpreted in terms of “safeguarding,” as smart teenagers are also highly motivated toward education achievements. Longitudinal research suggests that intelligence may interact with personality factors shaping mental health (Navrady et al., 2017). However, as far as we know there are no published data testing interactions between IQ and personality in shaping reproduction outcomes.

Finally, previous reports revealed gender differences in relations of personality and reproduction patterns. For example, Jokela et al. (2011) findings regarding the role of agreeableness and conscientiousness were valid only for females.

### The Present Study

Using Miller (1994) model as a frame, in the present study, using longitudinal data, we aimed at exploring possible links between personality traits and one reproductive success indicator, i.e., the number of children individuals have in early adulthood. We believe that the discovery of reproduction-related motives and traits is only possible when looking carefully at processes impacted by personality as early as possible in development. Following the results of Jokela et al. (2011) study we hypothesized that agreeableness and openness at age 12 would have a direct and positive association with the number of children in early adulthood. Based on the findings of Pearson et al. (2012) and Elder and MacInnis (1983) we hypothesized that age of first intercourse (AFI) and family and education plans would mediate the relation between childhood personality and the reproduction outcome. Following the results of Martin et al. (1977) we expected a negative association between extraversion and the AFI, and based on Jokela et al. (2011) findings we expected a positive relation between neuroticism and the AFI. Furthermore following the results provided by Elder and MacInnis (1983) we also expected a negative relation between neuroticism and familial plans and a positive relation of both neuroticism and conscientiousness with educational plans.

Following the findings of Retherford and Sewell (1989) and Halpern et al. (2000) we expected that IQ, measured at age 13, would be positively related to the age of first intercourse (i.e., higher IQ, later AFI), educational plans at age 17, and negatively to the number of one’s own children in early adulthood. We also hypothesized that IQ, as well as socioeconomic status and early stress experiences (based on Belsky et al., 1991 prediction) would interact with personality traits in the prediction of both mediators and the outcome variable of number of children. To test these hypotheses, we conducted a series of regression analyses. Furthermore, we used a regression model to explore potential moderator effects. We also built a structural equation model (SEM) incorporating the results of the first set of analyses.

Consistent with Jokela et al. (2011) we hypothesized that there would be gender differences in relations between personality and number of children (especially agreeableness and conscientiousness). To validate this hypothesis, we tested the equivalence of the SEM model in both genders. Because data on number of children were available only through age 27, we can test specific hypotheses regarding the relations of personality and reproductive patterns only in early adulthood.

## Materials and Methods

### Participants and Procedures

In the Child Development Project (CDP; Dodge et al., 1990) participants were recruited in two annual cohorts when entering kindergarten at three sites: Knoxville and Nashville, TN and Bloomington, IN, United States. Schools, where the recruitment process was performed, were identified based on federally subsidized lunch rates and neighborhood housing patterns to ensure inclusion of a high proportion of low-income children. During preregistration for kindergarten, parents were randomly picked and asked about their willingness to participate in a longitudinal study on child development. About 75% of parents agreed to participate. Because 15% of children did not preregister, that proportion of participants was recruited on the first day of school through letter or telephone (only participants who had not already been involved in the study were asked for participation) (Dodge et al., 1994). The sample consisted of 585 subjects (52% male) at the first assessment, representing mixed socioeconomic and ethnic background (81% were European American, 17% were African American, 2% belong to other ethnic groups; Hollingshead’s Index Mean = 38.74, SD = 13.2). Follow up assessments of participants or their parents were performed annually until age 27 through face-to- face interviews, telephone interviews, or questionnaire mail-outs. Please note that not all measures were applied annually, but only periodically (see details below). The data used in the current study were gathered between 1987 and 2009 (cohort I) or between 1988 and 2010 (cohort II). At the final assessment 82% of the original sample provided data. However, for some measures, data were obtained from a smaller number of participants (exact information for the particular scales is provided in the measures section). Until age 18 written consent was obtained from participants’ parents, and subsequently from the target participants themselves. The research protocols were approved by the institutional review boards at the universities associated with each site of the CDP.

### Measures

During year 1 of the CDP, mothers of the participants provided data on *gender* and *ethnicity* of the participants. Gender was coded as 0 = male, 1 = female, and ethnicity was coded as 0 = European American and other ethnic groups, 1 = African American.

Annually, between years 1 and 8, *stressful life events* (SLE) experienced by the participant or her/his family were assessed using the Changes and Adjustments Questionnaire (Dodge et al., 1994). Additionally, in the year 1 interview, mothers reported SLE from the child’s birth until age four. The CAQ consists of 18 items regarding different life events happening in the last year (or in the whole period before age 4), such as children’s accidents or injuries, severe or frequent illnesses, death(s) of family members, money or legal problems in the family, and parents’ divorce. Events were coded 0 (did not happen in the past year) or 1 (did happen in the past year). For each year the mean scores of life events were calculated. No indicator of internal consistency was calculated for this questionnaire because measured events can occur independently. Life events data were available for 69.2–99.1% of the sample at each time point (M = 82.8%). In the regression and SEM analyses, we used a regression-weighted composite measure of SLE based on factor loadings of each measurement.

*Socioeconomic status* of the participants was based on the Hollingshead Four-Factor Index of Social Status computed from parental education and occupation levels (scored ranging from 0 for unemployed to 8 for professional). The father’s data were included only when a biological father or another male partner of the mother lived at home. If no father lived at home, in line with Hollingshead’s recommendation, mothers’ data were double-weighted. In the current study, the SES indicator was based on the average across years when the child was in fifth through eighth grades.

*Personality traits* were measured by youth reports on a 25-item version of the Big Five Personality Questionnaire (BFPQ) (Lanthier, unpublished; Lansford et al., 2014) when participants were age 12, on 5-point scales (ranging from 1 = hardly at all to 5 = extremely much). The BFPQ is similar in content and structure to other self-report measures of personality during childhood (Barbaranelli et al., 2003). Each of the five scales has five items: Extraversion (α = 0.63), Agreeableness (α = 0.55), Conscientiousness (α = 0.63), Neuroticism (α = 0.58), Openness (α = 0.67). The validity of the BFPQ was provided by the longitudinal study of Fransson et al. (2013) on the stability of Big Five Personality factors. In the current study, personality data were obtained from 74% of the sample.

*IQ* was estimated based on the WISC Block Design and Vocabulary subtests (Sattler, 1988) which were administered as part of an interview when the participants were approximately 13 years old. IQ data were available for 75% of the sample.

*Familial and educational plans* were measured using items selected from the Career and Future Aspirations questionnaire, which was a part of an interview with the participants when they were 17 years old. Using a 5-point scale (ranging from 1 = very low to 5 = very high) they assessed the chance that a particular thing will happen. Familial and educational plans scores were calculated as a mean value of two items (“What are the chances that: You will be married, and you will have children” for familial plans, and “What are the chances that: You will graduate from high school, and you will go to college” for educational plans). Data were obtained for 76% of the sample.

At age 27 participants were asked about the *age* at which they *first* had *sexual intercourse* (AFI). The data from annual reports from ages 16 to 22 regarding whether they had sexual intercourse in the last year were also used if participants were missing data on the variable from age 27. During the same period, the participants answered the question on the *number of children* they have. The data were obtained for 85.5 and 78.8% of the sample, respectively.

### Analysis Plan

Due to partial lack of data, we first performed multiple imputation with 10 datasets (all missing data were at random). There were no statistical differences between participants with and without missingness in personality data for the outcome measures (*U* = 28392, *p* > 0.05 for the AFI and *U* = 16717.5, *p* > 0.05 for the number of children by age 27). As recommended by Hippel (2009) all interaction terms (except those based on the composite measure of SLE, which is a latent variable generated in structural equation modeling) were created before imputation. All variables constituting interaction terms were grand-mean centered. We performed imputation for gender groups separately. Second, we conducted a series of regressions to examine potential moderators of the relation between personality traits with the number of children and possible mediators of these links (i.e., the age of first sexual intercourse, familial and educational plans). Each regression included the main effect of the predictor, the main effect of the potential moderator, and the interaction term. IQ, SES, and the SLE composite measure were included as moderators. Third, to further explore relations between personality and the number of children, we performed a series of SEM analyses. The baseline model was created using the results of the regression analyses and theoretical predictions. Subsequent modifications of the baseline model were proposed through step-by-step procedures of removing non-significant paths. The best-fitting model was chosen on the basis of χ^2^ statistics. For the interpretation of model fit indices, we followed Hu and Bentler (1999) criteria. The indirect effects were tested using the bootstrapping method with 5,000 iterations. As the regression and SEM analyses were performed with the use of the same sample, the latter should be treated as confirmatory. Finally, we compared the obtained SEM model for boys and girls. All statistical analyses were conducted with the use of Mplus version 7 (Muthén and Muthén, 2012).

## Results

First, to identify the impact of personality traits we performed a series of regression analyses. These results are presented in the Supplementary Table 1. The results of initial correlation analysis and descriptive statistics are presented in Table 1.

**TABLE 1:** Descriptive statistics and bivariate correlations between variables (before imputation).

	Gender	Ethnicity	SES	SLE1_4	SLE5	SLE6	SLE7	SLE8	SLE9	SLE10	SLE11	SLE12	IQ	EDU	FAM	AFI
Gender	–	–														
Ethnicity	–	–														
SES	–0.06	−0.39**	–													
SLE1_4	0	–0.02	−0.13**	–												
SLE5	0.04	–0.04	−0.12**	0.39**	–											
SLE6	–0.07	0.02	−0.2**	0.31**	0.3**	–										
SLE7	−0.09*	–0.03	–0.09	0.21**	0.24**	0.39**	–									
SLE8	0.05	0.04	−0.16**	0.24**	0.15**	0.32**	0.3**	–								
SLE9	0	0.04	−0.15**	0.21**	0.14**	0.32**	0.28**	0.54**	–							
SLE10	0.04	0.11*	−0.2**	0.24**	0.2**	0.28**	0.3**	45**	0.39**	–						
SLE11	0.03	0.05	−0.16**	0.28**	0.19**	0.39**	0.26**	0.36**	0.39**	0.52**	–	.				
SLE12	0	–0.01	−0.13**	0.21**	0.12**	0.3**	0.25**	0.26**	0.39**	0.35**	0.51**	–				
IQ	−0.13**	−0.39**	0.41**	–0.03	0.02	−0.12*	–0.01	−0.1*	–0.06	−0.14**	−0.13**	–0.08	–			
EDU	0.06	−0.2**	0.32**	–0.09	–0.04	−0.15**	–0.09	–0.06	–0.06	−0.18**	–0.08	–0.05	0.36**	–		
FAM	0.09	−0.15**	0.11*	–0.05	–0.09	–0.08	−0.1*	–0.03	–0.03	–0.1	–0.04	–0.03	0.03	0.21**	–	
AFI	0.01	−0.14**	0.19**	−0.12**	–0.06	−0.14**	–0.08	−0.13**	–0.07	−0.15**	−0.12*	–0.09	0.24**	0.31**	–0.07	–
E	0.03	−0.13*	0.12*	0.01	0.06	0.04	0.06	0.01	0.02	0	–0.02	0.03	–0.03	0.01	0.08	−0.12*
A	0.02	0.01	–0.06	–0.02	0.06	–0.03	–0.02	–0.05	0.05	–0.01	–0.06	0.02	–0.05	0.01	0.08	–0.06
C	0.09	0.05	0.01	–0.05	0.02	–0.1	–0.04	–0.1	–0.08	–0.02	–0.03	–0.06	0.03	–0.03	0.14*	–0.03
N	0.18**	–0.01	–0.03	–0.01	0	0.02	–0.03	0.01	0.01	–0.01	0.05	–0.01	−0.17**	–0.05	–0.09	0.11*
O	−0.15**	0	0.16**	–0.05	–0.02	–0.07	0.04	–0.01	0.06	–0.09	–0.07	–0.02	0.29**	0.12*	–0.06	0.13*
ChN	0.13*	0.19**	−0.28**	0.14**	0.05	0.14**	0.03	0.15**	0.12*	0.19**	0.1	0.05	−0.35**	−0.38**	0.11*	−0.24**

		**E**		**A**		**C**		**N**		**O**			**ChN**		**M (SD)**	

Gender																–
Ethnicity																–
SES																38.74 (13.2)
SLE1_4																0.48 (0.27)
SLE5																0.28 (0.21)
SLE6																0.15 (0.12)
SLE7																0.16 (0.16)
SLE8																0.15 (0.14)
SLE9																0.14 (0.13)
SLE10																0.13 (0.12)
SLE11																0.14 (0.12)
SLE12																0.14 (0.11)
IQ																39.8 (9.43)
EDU																4.44 (0.93)
FAM																3.91 (0.97)
AFI																17.18 (3.22)
E	–															17.67 (3.36)
A	−0.15**	–														17.82 (2.68)
C	0.12*	0.24**	–													17.46 (3.12)
N	−0.36**	–0.09	–0.09	–												12.56 (3)
O	0.08	0.02	0.18**	−0.14**	–											18.12 (3.41)
ChN	–0.02	–0.06	0.04	0.09	−0.21**	–										0.77 (1.1)

SES, socioeconomic status; SLE, stressful life events; IQ, intelligence; EDU, educational plans; FAM, familial plans; AFI, age of first intercourse; E, extraversion; A, agreeableness; C, conscientiousness; N, neuroticism; O, openness; ChN, number of children. *p < 0.01, **p <0.001

We found main effects of neuroticism on the AFI and of conscientiousness on familial plans and the number of children by age 27. We also found a main effect of agreeableness on the number of children by age 27. Additionally, IQ had main effects on all outcome variables except familial plans, as did SES on all outcome variables except the AFI. SLE predicted the age of first intercourse and the number of children by age 27. We also found significant interaction effects between neuroticism and IQ on familial plans and the number of children by age 27, between openness and IQ and openness and SES on the AFI, and between agreeableness and SES on the number of children by age 27.

**FIGURE 1:**
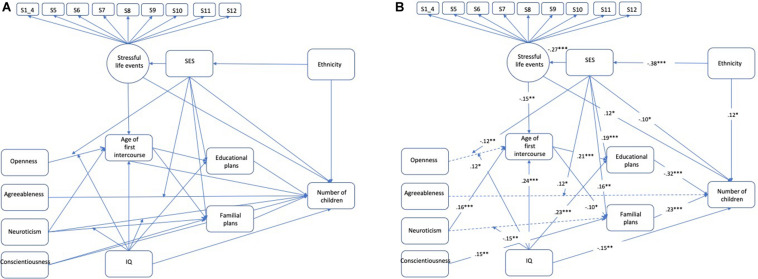
**(A)** The baseline model of relations among variables. S1_4 – stressful life events (SLE) reported from child’s birth until age four; S5 – S12 – SLE reported each year from age five until age twelve; SES – socioeconomic status, All error terms and correlation paths for personality traits and stressful life events were omitted for clarity. **(B)** The final model of relations among variables. All abbreviations as in panel **A**; ^∗^*p* < 0.05;^∗∗^*p* < 0.01; ^∗∗∗^*p* < 0.001; Paths with dashed lines are non-significant; All error terms and correlation paths for personality traits and stressful life events were omitted for clarity.

Second, using theoretical predictions and results of regression analyses, we constructed a structural model. Due to probable overlap across years in stressful events, we allowed for correlations between error terms of neighboring SLE measurements. The baseline model is presented in Figure 1A and the final model (with all modifications) in Figure 1B.

The fit statistics of the baseline model were satisfactory and are presented in Table 2. However, we decided to remove non-significant paths step-by-step to improve the fit of the model. First, we removed the path from the AFI to the number of children by age 27. The resulting model (2) fit well and its comparison with model 1 using the chi-square difference was non-significant, Δχ*^2^*(*df* = 1) = 0.5, *p* > 0.05, so we chose the more parsimonious model 2. Next, we removed the path from Conscientiousness to the number of children by age 27. The resulting model’s fit was better than model 2. The comparison of models 2 and 3 was non-significant (Δχ*^2^*(*df* = 1) = 0.3, *p* > 0.05), so model 3 was preferred. Finally, we removed the interaction of Neuroticism and IQ on the number of children by age 27. The obtained model 4 fit as well as model 3 – (Δχ*^2^*(*df* = 2) = 2.05, *p* > 0.05), so model 4 was the final model. Although the fit statistics for the final model were satisfactory, two of them (CFI and TLI) did not reach Hu and Bentler (1999) criteria. Nevertheless, fit indexes still exceed conventional criteria for a good fit, and we note Kenny (2014) suggestion that incremental fit indices such as CFI and TLI may not be fully informative if the RMSEA for the null model is less than 0.158. Null RMSEA for all tested models in our study was 0.092.

**TABLE 2:** Comparison between different models - model fit statistics.

Model	χ^2^	*df*	RMSEA	SRMR	CFI	TLI
Model 1 (baseline)	293.6**	207	0.027 (0.019–0.034)	0.054	0.924	0.915
Model 2 (Model 1 without the AFI – number of children path)	294.1**	208	0.027 (0.019–0.033)	0.054	0.924	0.916
Model 3 (Model 2 without Conscientiousness – number of children path)	294.43**	209	0.026 (0.019–0.033)	0.054	0.925	0.917
Model 4 (Model 3 without N × IQ – number of children path)	296.48**	211	0.026 (0.019–0.033)	0.054	0.925	0.918
Model 4/females	289.96**	211	0.036 (0.025–0.046	0.078	0.872	0.86
Model 4/males	261.09*	211	0.028 (0.014–0.039)	0.068	0.911	0.902
Model 4 females vs. males unconstrained	569.33**	438	0.032 (0.024–0.039)	0.074	0.888	0.882
Model 4 females vs. males constrained	614.97**	472	0.032 (0.025–0.039)	0.079	0.878	0.881

AFI, Age of first intercourse; N, Neuroticism; RMSEA, the root mean square error of approximation; SRMR, the standardized root mean residual; CFI, confirmatory fit index; TLI, the Tucker-Lewis index; the 90% CI for RMSEA are in brackets; *df*, degrees of freedom; *p <0.05; **p < 0.001

**FIGURE 2:**
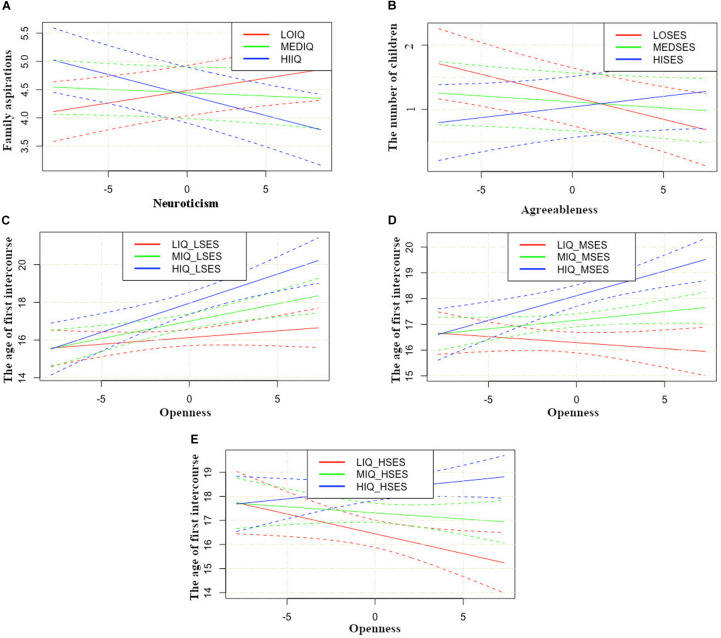
**(A)** Plot of interaction of Neuroticism with IQ on Family Aspirations. LOIQ, low IQ group; MEDIQ, medium IQ group; HIIQ, high IQ group. Personality traits measures are presented as standardized values. Low, medium and high values of IQ were picked up as –1SD, mean and +1SD. **(B)** Plot of interaction of Agreeableness with SES on the number of children. LOSES, low SES group; MEDSES, medium SES group; HISES, high SES group. Personality traits measures are presented as standardized values. Low, medium and high values of SES were picked up as –1SD, mean and +1SD. **(C)** Plot of interaction of Openness with IQ on the age of first intercourse in low SES group. LIQ, low IQ group; MIQ, medium IQ group; HIQ, high IQ group. Personality traits measures are presented as standardized values. Low, medium and high values of IQ were picked up as –1SD, mean and +1SD. **(D)** Plot of interaction of Openness with IQ on the age of first intercourse in medium SES group. **(E)** Plot of interaction of Openness with IQ on the age of first intercourse in high SES group. LIQ, low IQ group; MIQ, medium IQ group; HIQ, high IQ group.

The interaction effects in the SEM model are represented in Figures 2A–E.

First, Figure 2A shows that the impact of neuroticism on Family plans is moderated by IQ, with neuroticism positively predicting family plans for those in the low IQ group and negatively for those in the high IQ group. Second, Figure 2B shows a negative relation of agreeableness to the number of children by age 27 in the low SES group, and a positive one in the high SES group. Third, the 3-way interaction depicted in Figures 2C–E shows that the impact of openness on AFI was moderated both by IQ and SES. There was a positive relation between openness and AFI for individuals with high IQ in all three SES groups. There was also a negative relation between openness and AFI for individuals with low IQ in the high SES group.

We also checked for indirect effects of personality traits and potential mediators on the number of children by age 27. There was a significant negative impact of neuroticism through the AFI and Educational plans, and through Familial plans but only for individuals with high IQ. We also identified the indirect positive impact of conscientiousness through Familial plans. Additionally, there was a negative indirect effect of openness through the AFI and Educational plans but only for individuals with low SES and medium or high IQ. A similar pattern was also observed for individuals with both medium SES and high IQ. IQ was related indirectly to the number of children by age 27 through the AFI and Educational plans and through Educational plans alone. The indirect effect of SES was detected through SLE, the AFI, and Educational plans, through Educational plans alone, and through Familial plans. Stressful life events were related indirectly to the number of children through the AFI and Educational plans. All standardized coefficients for indirect and total effects are presented in Table 3.

**TABLE 3:** Standardized coefficients for indirect and total effects.

Path	Condition	Beta	SE
Neuroticism through the AFI and Educational plans		−0.01**	0.1
Neuroticism through the AFI and Familial plans		0	0
Neuroticism through Familial plans	(low IQ)	0.03	0.03
	(medium IQ)	–0.01	0.01
	(high IQ)	−0.06**	0.02
Neuroticism – total effect		–0.07	0.05
Conscientiousness through Familial plans		0.04**	0.01
Openness through the AFI and Familial plans	(low IQ, low SES)	–0.01	0.01
	(medium IQ, low SES)	–0.01	0.01
	(high IQ, low SES)	–0.01	0.01
	(low IQ, medium SES)	0	0
	(medium IQ, medium SES)	0	0
	(high IQ, medium SES)	0	0
	(low IQ, high SES)	0	0
	(medium IQ, high SES)	0	0
	(high IQ, high SES)	0	0
Openness through the AFI and Educational Plans	(low IQ, low SES)	–0.01	0.01
	(medium IQ, low SES)	−0.02*	0.01
	(high IQ, low SES)	−0.03*	0.01
	(low IQ, medium SES)	0	0
	(medium IQ, medium SES)	0	0
	(high IQ, medium SES)	−0.02*	0.01
	(low IQ, high SES)	0.02	0.01
	(medium IQ, high SES)	0.01	0.01
	(high IQ, high SES)	–0.01	0.01
Openness – total effect	(low IQ, low SES)	–0.01	0.01
	(medium IQ, low SES)	−0.02*	0.01
	(high IQ, low SES)	−0.04**	0.01
	(low IQ, medium SES)	0	0.01
	(medium IQ, medium SES)	–0.01	0.01
	(high IQ, medium SES)	−0.02*	0.01
	(low IQ, high SES)	0.02	0.01
	(medium IQ, high SES)	0.01	0.01
	(high IQ, high SES)	–0.01	0.01
	(low SES)	−0.07*	0.03
	(medium SES)	–0.03	0.02
	(high SES)	0.02	0.03
	(low IQ)	0.01	0.03
	(medium IQ)	–0.03	0.02
	(high IQ)	−0.07*	0.03
	(all conditions)	–0.08	0.06
IQ through the AFI and Educational Plans		−0.02*	0.01
IQ through the AFI and Familial Plans		–0.01	0
IQ through Educational Plans		−0.09**	0.03
IQ – total effect		−0.29***	0.07
SES through the SLE, the AFI and Educational Plans		−0.01*	0.01
SES through the SLE, the AFI and Familial Plans		0	0
SES through Educational Plans		−0.07**	0.02
SES through Familial Plans		0.04*	0.02
SES – total effect		−0.13*	0.06
SLE through the AFI and Educational Plans		0.01*	0.01
SLE through the AFI and Familial Plans		0	0
SLE – total effect		0.17*	0.07

AFI, Age of first intercourse; SLE, stressful life events; SES, socioeconomic status; *p < 0.05; **p < 0.01; ***p < 0.001.

Finally, we tested the possible moderating role of gender. The final model adequately fits in both groups (as shown in Table 2). Thus, we performed a fit analysis of unconstrained and constrained final models. The fit statistics are satisfactory and shown in Table 2. The comparison of both models using the chi-square difference was non-significant −Δχ*^2^*(*df* = 34) = 45.64, *p* > 0.05. This finding implies that the path coefficients across groups are equal, and gender does not moderate the relations between variables.

## Discussion

### Personality, Number of Children, and Goals

We found a rich pattern of relations between personality traits in late childhood and the number of children by age 27. Each Big 5 personality variable, except extraversion, had either a direct or indirect relation with number of children by age 27. Previous studies using extraversion or similar traits as a predictor of number of children have shown mixed results (Nettle, 2005). Importantly, previous research on this topic measured personality in late adolescence at the earliest. In our study personality assessment was made during late childhood/early adolescence. Although overall extraversion seems to be stable across development, some of its facets, especially those related to sociability, increase during early adolescence (Durbin et al., 2015). Hence, it is possible that we were not able to capture the possible influence of extraversion.

Unexpectedly, we did not find a relation of conscientiousness with educational goals. However, we did find a positive relation of conscientiousness with familial plans. Conscientiousness is related to adherence to social norms. It seems possible that the association between conscientiousness and plans for a family reflects the adherence to the norm of building a family. Additionally, it is worth noting that the impact of conscientiousness on educational aspirations is reduced after controlling for other factors (Rottinghaus et al., 2002). It is possible that due to relations between conscientiousness and IQ (Luciano et al., 2006) the effect of this personality trait on educational goals in our study was reduced.

### The Importance of Neuroticism

As expected, we found a positive relation between neuroticism and the age of first intercourse—that is, delayed sexual intercourse—which appears to support the Blinn-Pike et al. (2004) finding that some adolescents delay intercourse due to fear and embarrassment about sex. We also found a negative association of neuroticism with familial plans. We did not find a direct relation between neuroticism and educational plans. We found that late onset of sexual activity was positively related to higher educational plans and predicted fewer children by age 27, and that this course of events is partially related to neuroticism. The lower number of children is probably a consequence of putting education goals over family plans. This mechanism is potentially influenced by neuroticism. However, the negative relation between neuroticism and familial plans resulting in fewer children by age 27 is only found for individuals with high IQ. A combination of high neuroticism and high IQ may have a protective (or positive) impact as shown in the context of mental health problems risk (Navrady et al., 2017).

### The Protective Role of High IQ

Together with findings showing direct and indirect (mainly through educational plans) influence of IQ on the number of children by age 27, these data support the “safe-guarding” hypothesis. The postponement of sexual intercourse (and consequently fewer children by age 27) may be interpreted as a manifestation of adolescents’ desires to protect their future educational plans by avoiding the risk related to intercourse. We can interpret this relation in terms of Hirschi (1969) social control theory. Cognitively able children will be more likely to receive reward at school and consequently develop ambitious educational and occupational plans. This protective role of high IQ is clearly seen when we look at its interaction with openness on the age of first intercourse. We found that openness at age 12 is positively related to AFI for children with high IQ in all SES groups. However, we also found a negative relation between openness and AFI for children with low IQ and high SES. A possible explanation for this might be the existence of the gap between individuals’ cognitive abilities and environmental expectations, which leads to resignation from the typical goals of high SES youths, along with engagement in risky and unconventional behaviors. An analogous effect was observed and similar explanation was proposed for the higher risk of obesity (Goldberg et al., 2013).

### SES and Risky Behaviors

Some research has shown that adolescents from high SES groups are more likely to engage in risky behaviors than youths from other SES groups (Racz et al., 2011). The level of perceived stress may partially explain the relation between SES and risky behaviors. Lee and Seo (2018) found that heightened stress in high SES youths engaging in unconventional and risky behaviors stems from the environmental pressure on academic achievements. We may speculate that this effect would be dramatically magnified in children having both low IQ and high SES. However, this needs to be further explored. We must note here that we did not observe a direct relation of openness with the number of children by age 27, but we did find an indirect negative association for specific IQ and SES conditions. As hypothesized, we found a relation between agreeableness and the number of children by age 27. However, parental SES moderated this association. Our findings partially overlap with the results of Jokela et al. (2011) who found a positive relation between agreeableness and the number of children, but only in females. Low agreeableness is related to sexual risk taking behaviors (e.g., unprotected sex) (Hoyle et al., 2000) but high SES may act as a buffer against these tendencies. Nevertheless, the overall impact of SES and ethnicity in our study was as expected from previous studies (Sweeney and Raley, 2014). As hypothesized we also found indirect and direct effects of stressful life events on the number of children (Belsky et al., 1991). Together, these findings support Miller (1994) assumption of amalgamation of biologically founded traits with life experiences in the formation of specific reproduction pathways. Future studies should focus on identifying particular cognitive mediators of this transformation.

### Gender as a Moderator

We found that gender did not moderate the effects identified in the SEM. Previous studies (Jokela et al., 2011) revealed gender-specific relations between personality traits and reproductive outcomes. However, there are considerable differences between this study and the previous ones. First, the number of children in our study was assessed at age 27, but many participants had not yet had children by this age. Second, we used self-reported measures of personality, which were administered in late childhood, whereas previous studies used data gathered in adult samples.

## Limitations

The present study has some limitations. First, personality scales have low-reliability coefficients. The primary source of this problem stems from the length of these scales (each has only five items). However, as noted by Ortet et al. (2012) several studies of adolescents using scales based on the big five theory constructs found reliability problems. They speculate on possible factors influencing the assessment limitations pointing at personality developmental trends or difficulties in verbal comprehension described in children. Regardless of the mentioned factors, low reliabilities of the personality scales in the present study may affect the detected relations between personality and other variables. Second, our study did not include a measurement of personality changes through adolescence and early adulthood. Some research (Neyer and Lehnart, 2007) showed that individual differences in personality change during this period might influence the processes of the formation of close relationships and, consequently, as we believe, family plans. Third, in Western societies, the age of 27 is not typically considered as an age for planning to have children. Hence, the results of our study must not be viewed in the context of the total number of children of an individual but should instead be regarded in terms of factors influencing early parenthood. Moreover, we have to remember that cultural factors strongly influence the processes of family planning. Fourth, we did not control for many other socio-economic variables, which may potentially impact decisions related to having a family. Fifth, the age of first intercourse was assessed retrospectively, which may be a source of unreliability (Upchurch et al., 2002). Future longitudinal studies should incorporate alternate ways of measuring age of first intercourse.

## Conclusion

In conclusion, using data from a longitudinal cohort we were able to model relations between late childhood personality and age of first intercourse and the number of children by age 27. These associations are mediated by adolescents’ plans related to family formation and education as well as their sexual activity, and are moderated by socio-economic characteristics.

## Data Availability Statement

The datasets generated for this study are available on request to the corresponding author.

## Ethics Statement

The studies involving human participants were reviewed and approved by the Institutional review board, Indiana University; Institutional review board, Auburn University; Institutional review board, Duke University. Written informed consent to participate in this study was provided by the participants’ legal guardian/next of kin.

## Author Contributions

JB, KD, and GP contributed to the conception and design of the study, acquired funding sources, and managed data collection. WD performed the statistical analyses and wrote the first draft of the manuscript. JL helped to manage the data collection and organized the database. WD, JL, and JB wrote the sections of the manuscript. All authors contributed to the manuscript revision, read and approved the submitted version.

## Conflict of Interest

The authors declare that the research was conducted in the absence of any commercial or financial relationships that could be construed as a potential conflict of interest.
