# Supplementary material for: Individual and Environmental Predictors of Age of First Intercourse and Number of Children by Age 27
Source: Front Psychol. 2020 Jul 8;11:1639. doi: 10.3389/fpsyg.2020.01639 (PMC7362713; doi:10.3389/fpsyg.2020.01639)
Supplement: Supplementary file 1 [file Table_1.docx]

Supplementary Table 1. Results of series of regression analyses (unstandardized coefficients)

| Predictors | Predicting age of first intercourse | | Predicting familial plans | | Predicting educational plans | | Predicting number of children | |
| --- | --- | --- | --- | --- | --- | --- | --- | --- |
|  | B | SE | B | SE | B | SE | B | SE |
| Extraversion (E) | -.08 | .05 | .01 | .02 | 0 | .01 | -.01 | .02 |
| Agreeableness (A) | 0 | .06 | -.01 | .02 | .01 | .02 | -.04* | .02 |
| Conscientiousness (C) | -.01 | .06 | .05** | .02 | -.01 | .02 | .04* | .02 |
| Neuroticism (N) | .14** | .05 | -.03 | .02 | -.01 | .02 | 0 | .02 |
| Openness (O) | .08 | .05 | -.03 | .02 | .01 | .02 | -.03 | .02 |
| Stressful life events (SLE) | -4.7** | 1.6 | -.66 | .51 | -.72 | .46 | 1.38* | .56 |
| Intelligence (IQ) | .07*** | .02 | 0 | .01 | .02*** | .01 | -.03*** | .01 |
| Socioeconomic status (SES) | .02 | .01 | .01* | .01 | .01** | .01 | -.01* | .01 |
| ExSLE | .19 | .53 | .13 | .16 | .13 | .18 | -.03 | .16 |
| AxSLE | -.5 | .56 | .11 | .21 | .08 | .18 | .01 | .26 |
| CxSLE | -.07 | .47 | .09 | .18 | -.22 | .15 | .3 | .19 |
| NxSLE | -.4 | .57 | .38 | .2 | .29 | .17 | -.18 | .2 |
| OxSLE | -.27 | .47 | .02 | .16 | .12 | .18 | -.21 | .17 |
| ExIQ | -.01 | .01 | 0 | 0 | 0 | 0 | 0 | 0 |
| AxIQ | -.01 | .01 | 0 | 0 | 0 | 0 | 0 | 0 |
| CxIQ | 0 | .01 | 0 | 0 | 0 | 0 | 0 | 0 |
| NxIQ | 0 | .01 | -.01* | 0 | 0 | 0 | -.01** | 0 |
| OxIQ | .01* | .01 | 0 | 0 | 0 | 0 | 0 | 0 |
| ExSES | 0 | .01 | 0 | 0 | 0 | 0 | 0 | 0 |
| AxSES | 0 | .01 | 0 | 0 | 0 | 0 | .01* | 0 |
| CxSES | 0 | .01 | 0 | 0 | 0 | 0 | 0 | 0 |
| NxSES | 0 | 0 | 0 | 0 | 0 | 0 | 0 | 0 |
| OxSES | -.01* | .01 | 0 | 0 | 0 | 0 | 0 | 0 |

**p* < .05; ***p* < .01; ****p* < .001; SLE – composite measure of stressful life events
